# Aceruloplasminemia: Waiting for an Efficient Therapy

**DOI:** 10.3389/fnins.2018.00903

**Published:** 2018-12-04

**Authors:** Alberto Piperno, Massimo Alessio

**Affiliations:** ^1^Department of Medicine and Surgery, University of Milano-Bicocca, Monza, Italy; ^2^Medical Genetic Unit, San Gerardo Hospital, ASST-Monza, Monza, Italy; ^3^Division of Genetics and Cell Biology, IRCCS-Ospedale San Raffaele, Milan, Italy

**Keywords:** aceruloplasminemia, neurodegeneration, iron overload, ferroxidase, diabetes, ceruloplasmin, iron chelation, plasma

## Abstract

Aceruloplasminemia is an ultra-rare hereditary disorder caused by defective production of ceruloplasmin. Its phenotype is characterized by iron-restricted erythropoiesis and tissue iron overload, diabetes, and progressive retinal and neurological degeneration. Ceruloplasmin is a ferroxidase that plays a critical role in iron homeostasis through the oxidation and mobilization of iron from stores and subsequent incorporation of ferric iron into transferrin (Tf), which becomes available for cellular uptake via the Tf receptor. In addition, ceruloplasmin has antioxidant properties preventing the production of deleterious reactive oxygen species via the Fenton reaction. Some recent findings suggest that aceruloplasminemia phenotypes can be more heterogeneous than previously believed, varying within a wide range. Within this large heterogeneity, microcytosis with or without anemia, low serum iron and high serum ferritin, and diabetes are the early hallmarks of the disease, while neurological manifestations appear 10–20 years later. The usual therapeutic approach is based on iron chelators that are efficacious in reducing systemic iron overload. However, they have demonstrated poor efficacy in counteracting the progression of neurologic manifestations, and also often aggravate anemia, thereby requiring drug discontinuation. Open questions remain regarding the mechanisms leading to neurological manifestation and development of diabetes, and iron chelation therapy (ICT) efficacy. Recent studies in animal models of aceruloplasminemia support the possibility of new therapeutic approaches by parenteral ceruloplasmin administration. In this review we describe the state of the art of aceruloplasminemia with particular attention on the pathogenic mechanisms of the disease and therapeutic approaches, both current and perspective.

## Introduction

Aceruloplasminemia (ACP) was firstly described in 1987 as an autosomal recessive disease caused by an inactivating mutation of the ceruloplasmin gene (CP) (Miyajima et al., 1987). The rarity of the disease (estimate prevalence about 1:2,000,000) is a major limit for a comprehensive definition of phenotype, genotype-phenotype associations, therapeutic efficiency, and development of disease markers and drugs. Indeed, the typical manifestations make ACP a unique iron overload disease being: (i) The only one among the Neurodegeneration with Brain Iron Overload disorders, to whom ACP belongs, manifesting systemic iron overload; (ii) The unique systemic iron overload disease characterized by neuropathy as major cause of morbidity and microcytic anemia with low serum iron and transferrin (Tf) saturation as a common manifestation. This phenotype is related to the function of ceruloplasmin (Cp) in iron homeostasis at both systemic and brain levels. The lack of Cp induces cellular iron retention and progressive overload on the one hand, and low cellular iron release leading to iron-restricted erythropoiesis and iron deficiency anemia, on the other hand. However, the physiopathology of organ damage, as well as the pathways and efficiency of chelators in brain iron removal, are highly controversial issues which remain to be fully elucidated.

## Clinical Heterogeneity of Aceruloplasminemia

Recent studies showed that ACP phenotypes are more heterogeneous than previously believed and that some manifestations may precede neurological manifestation even by decades (Pelucchi et al., 2018). Thus, this could allow early diagnosis, hopefully preventing the development of neurological derangements, which are the most severe complication of this disease. Neurological symptoms usually appear in the fifth decade of life and vary within a wide spectrum that includes cerebellar ataxia, involuntary movements, parkinsonism, mood and behavior disturbances, and cognitive impairment (McNeill et al., 2008; Kono, 2012). As magnetic resonance imaging (MRI) is increasingly used in neurological diagnostics, brain iron accumulation can be found in a few patients with neuro-psychiatric symptoms addressing diagnosis to ACP. However, ACP patients without brain iron overload or patients with brain iron overload without or with only very mild neurological manifestations even after 50 years of age have been reported (Skidmore et al., 2008; Kassubek et al., 2017; Pelucchi et al., 2018), suggesting that genetic and/or acquired factors may partially modify neurologic phenotype.

Retinal manifestations are reported in over 75% of Japanese patients (Miyajima, 1993; Kono, 2012), but these were less frequent in Italian patients (Pelucchi et al., 2018). However, accurate descriptions of retinopathy are available in only two patients (He et al., 2007) ranging from retinal discoloration to macular degeneration, indicating that further studies are needed to better characterize ACP-dependent retinopathy.

Diabetes mellitus is considered an early manifestation of ACP, being reported as the first symptom in 68.5% of patients at a median age of 38.5 years in a recent overview. Diabetes was insulin dependent in 60% of patients, undefined in 30%, and less than 10% orally and/or dietary treated (Vroegindeweij et al., 2015). Little information is available on family susceptibility, concomitant overweight, and beta-cells function, which would be useful in understanding diabetes pathogenesis in ACP.

Often not included in the classical triad of ACP (diabetes, retinopathy, and neuropathy), anemia and/or microcytosis are common manifestations being reported in 80% of ACP patients in Japan (Miyajima, 1993) and recorded as the earliest manifestations of disease in 86% of Italian patients, preceding diagnosis even by decades (Pelucchi et al., 2018). However, in a retrospective review of reported cases, anemia was recorded as the first symptom only in 24% of patients (Vroegindeweij et al., 2015). The high frequency of mild anemia and/or microcytosis in the general population, which are likely to be related to iron deficiency or thalassemia traits, can lead to the underestimation of these signs as early ACP-related manifestations. Thus, despite ACP rarity, Cp measurement should be included in the diagnostic work-up of anemia. The presence of low serum iron and Tf saturation with normal/high serum ferritin may serve to further enhance disease suspicion.

## Physiopathology of Aceruloplasminemia

**FIGURE 1:**
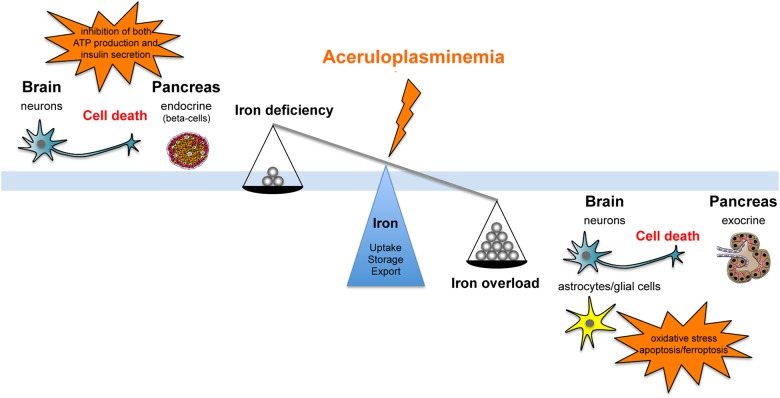
Schematic representation of the effect that aceruloplasminemia can promote on iron balance either fostering iron overload or iron deficiency, inducing neurons and islet beta-cells distress due to elevated sensitivity/vulnerability to iron toxicity or to iron deficient status consequent to metal retention in the surrounding cells.

Ceruloplasmin is part of the multicopper ferroxidase family, a group of tissue specific proteins (Cp, hephaestin, and zyklopen) that facilitate cellular iron efflux in conjunction with the membrane ferrous iron exporter ferroportin (Fpn), by oxidizing ferrous iron to the ferric state (Vashchenko and MacGillivray, 2013). Cp is recognized as a serum protein secreted by the liver, but it has also been found as glycosylphosphatidylinositol (GPI)-linked protein in astrocytes and leptomeningeal cells in the central nervous system (CNS), but also in macrophages, hepatocytes and many other tissues (Musci et al., 2014). Cp-KO mice revealed an impairment in hepatocyte and reticuloendothelial iron efflux (Harris et al., 1999), and showed that Cp-GPI is relevant for brain iron metabolism in regulating iron efflux from astrocytes (Jeong and David, 2003). A molecular connection between Cp and Fpn has been established with the finding that ferroxidase activity is required to stabilize Fpn at the cell surface in cells expressing Cp-GPI (De Domenico et al., 2007). Thus, Cp can be considered as a second determinant of Fpn stability after hepcidin. These findings suggest that differently from most disorders of iron overload that reflect changes in the absolute amount of iron, ACP results, at least in early stages, from iron imbalance caused by impairment in the rate of iron efflux from storage sites. Serum hepcidin is decreased in ACP patients, possibly related to the low Tf saturation, suggesting that suppressed hepcidin synthesis might contribute to the development of iron overload (Kaneko et al., 2010). While it is clear that microcytosis, anemia and low serum iron depend on reduced iron availability due to cellular iron sequestration, the development of diabetes and neurodegeneration in ACP are not evidently explained at cellular and molecular levels. It has been suggested that both diabetes and neurodegeneration are secondary to iron accumulation and toxicity (Kono, 2012), however, no clear evidence of intracellular iron deposition in the endocrine pancreas and in neurons has been reported in neither ACP patients nor mice models of ACP (Kato et al., 1997; Jeong and David, 2006; Chen et al., 2018). These findings suggest that neurons and islet beta-cells distress might be related to an elevated sensitivity/vulnerability to iron toxicity or to the iron deficient status induced by iron retention in the surrounding cells (Figure 1).

### Physiopathology of Brain Iron Overload and Damage

Iron enters the CNS via the brain barrier systems, namely the blood brain barrier (BBB) and the blood-cerebrospinal fluid barrier (BCSFB), which separate the blood from the interstitial- and cerebrospinal-fluid (CSF), respectively. The mechanism of iron uptake/export in cells depends on the iron transport molecules they express. Transferrin-bound-iron, internalized by transferrin receptor 1 (TfR1) in brain capillary endothelial cells (BCECs), can cross the BBB by: (1) transcytosis and direct release as ferric iron in the brain parenchyma, or (2) iron release from endosomes to cytosol via divalent metal ion transporter 1 (DMT1), and exported via Fpn assisted by the GPI-Cp expressed on perivascular astrocytes (McCarthy and Kosman, 2015; Simpson et al., 2015). The absence of iron accumulation in BCECs in both ACP patients and murine models indicate transcytosis and direct iron release as the more plausible mechanism in ACP (Miyajima, 1993; Zanardi et al., 2018). The BCSFB comprises choroid plexus epithelial cells (CPEpiCs) that are in contact with the CSF on one side, and with fenestrated endothelial cells on the other. In CPEpiCs iron is internalized via TfR1 and then exported to the CSF by the action of Fpn and Cp, which are both expressed by CPEpiCs (Rouault et al., 2009; Zheng and Monnot, 2012). Large iron deposition in CPEpiCs precedes accumulation in other brain regions in Cp-KO mice (Zanardi et al., 2018) suggesting an early deficit in iron handling by these cells in ACP. However, the pathophysiology of choroid plexus damage in ACP patients is largely undefined.

Neurons acquire iron prevalently from Tf and TfR1 while oligodendrocytes and astrocytes do not express TfR1, which suggests they import iron using other mechanisms (Zhang et al., 2006; Belaidi and Bush, 2016). Astrocytes may acquire iron released by BCECs being in close contact with them via endfeets that highly express DMT1 (McCarthy and Kosman, 2015). However, DMT1 uptake requires ferric iron to be reduced, but the mechanism of iron reduction by astrocytes is still unclear. Oligodendrocytes and astrocytes are rich in ferritin, suggesting that they function as iron storage site for the whole brain (Finazzi and Arosio, 2014). Regardless of import mechanisms, iron efflux from all brain cells involve Fpn, and depends on the extracellular ferroxidase activity fostered primarily by both the membrane Cp-GPI and the soluble Cp circulating in the interstitial fluid and CSF (Patel and David, 1997; Olivieri et al., 2011). Therefore, in ACP, the absence of Cp-ferroxidase activity promotes Fpn internalization and degradation favoring intracellular iron accumulation (Kono et al., 2010; Persichini et al., 2012).

Brain iron accumulation, particularly in the basal ganglia and cerebellum, is reported by MRI in a large number of ACP patients (Grisoli et al., 2005; Kono, 2012). Nevertheless, the few autopsy studies performed indicated that iron deposition was more evident in glial and astrocytes than in neurons, in particular in the perivascular regions of brain areas where neuronal loss is prevalent (Kawanami et al., 1996; Kaneko et al., 2002, 2012; Oide et al., 2006). Thus, it has been hypothesized that apoptosis/ferroptosis of iron-engulfed astrocytes/glial cells might release large amounts of toxic ferrous iron promoting oxidative neuronal damage, as inferred by large lipids peroxidation observed in the brain of ACP patients (Miyajima et al., 1998, 2001a; Kohno et al., 2000; Yoshida et al., 2000). Neuronal damage could be aggravated by the loss of glial-derived growth factors necessary for neuronal survival (Jeong and David, 2006). It is also conceivable that neuronal injury might depend on iron deficiency as the consequence of impaired mobilization of the accumulated iron in glial cells (Jeong and David, 2006; Kono, 2013).

### Physiopathology of Systemic Iron Overload and Damage

In the liver of ACP patients, iron accumulation prevails in hepatocytes, a surprising finding considering the role of Cp in regulating cellular iron efflux via Fpn. (Morita et al., 1995; Kawanami et al., 1996; Hellman et al., 2000; Bosio et al., 2002; Loreal et al., 2002; Finkenstedt et al., 2010; Rusticeanu et al., 2014). Indeed, liver iron distribution differs in patients with classical Fpn disease and ACP, being prevalent in reticuloendothelial cells in the former and in hepatocytes in the latter, indicating the need to expand our knowledge on the relative function of Fpn and Cp and their interactions in different tissues. Despite hepatocellular iron overload, cirrhosis is uncommon in ACP, however, some patients developed moderate/severe fibrosis in the presence of heavy hepatic iron overload (Pelucchi et al., 2018). This suggests that in ACP patients the risk of liver fibrosis depends on the iron burden, a similar scenario to hereditary hemochromatosis (HH) (Wood et al., 2008), and that liver iron overload in ACP patients generally does not reach the threshold associated with liver damage risk.

Clinically, diabetes may precede neurological derangements even by decades, but the mechanism of endocrine pancreatic damage in ACP is unclear. Iron accumulation in the exocrine pancreas has been commonly reported in mouse models characterized by systemic iron loading (hypotransferrinemic mice, bone morphogenetic protein 6, hemojuvelin-, and hepcidin-deficient strains) (Chen et al., 2018). About 20% of HH patients show diabetes, all of them being heavy iron overloaded and the majority having liver fibrosis/cirrhosis (McClain et al., 2006). In HH-patients iron deposition is present in the exocrine pancreas and, varied from case to case, in beta-cells together with loss of endocrine granules (Rahier et al., 1987). In mouse models of HFE-HH, iron accumulates specifically in the endocrine pancreas resulting in increased beta-cell death (Cooksey et al., 2004). The total pancreatic- and per islet-insulin content were low in Hfe-KO mice (Huang et al., 2011), suggesting an iron-related beta-cell oxidative stress and decreased insulin secretory capacity secondary to beta-cell apoptosis and desensitization of glucose-induced insulin secretion (Backe et al., 2016). However, in other mouse models of hemochromatosis, the endocrine pancreas was unaffected. Hepcidin-KO mice developed chronic pancreatitis owing to exocrine iron overload (Lunova et al., 2017), and, similarly, a murine model of Fpn mutant resistant to hepcidin binding showed exocrine pancreatic failure and iron overload (Altamura et al., 2014). Iron deposition and functional deficit restricted to the exocrine pancreas has also been reported in the double Cp/Hephaestin-KO mice model that did not display structural or functional (insulin secretion) deficiency of pancreatic islets (Chen et al., 2018).

Few studies describe pancreatic alteration in ACP patients mainly reporting generic iron accumulation without histological description of the cells and structures involved (exocrine vs. endocrine) (Daimon et al., 1995; Kawanami et al., 1996). Three reports described the absence or mild iron accumulation in the islets compared to the exocrine pancreas (Morita et al., 1995; Kato et al., 1997; Miyajima et al., 2001b). In a single ACP patient, an autopsy study showed marked reduction of insulin-containing cells without iron accumulation and degeneration/loss of islet cells, despite massive iron deposition in the exocrine pancreas (Kato et al., 1997). The patient’s long type-2 diabetes history may suggest beta-cell exhaustion as a possible explanation for such findings. Nevertheless, the pathogenesis of diabetes in ACP and the role of iron remain elusive, also considering that diabetes can occur early when systemic iron overload is still minor (Hatanaka et al., 2003; Muroi et al., 2006; Pelucchi et al., 2018).

## Treatment of Aceruloplasminemia

Current treatment in ACP (Table 1) is primarily focused on reducing iron overload using iron chelators (deferasirox, deferoxamine, and deferiprone). In the majority of patients, iron chelation therapy (ICT) was effective in reducing systemic iron overload, but there is no information on whether it can improve glucose metabolism derangement and retinopathy. ICT was less or not effective on neurological symptoms [reviewed in (Kono, 2013; Dusek et al., 2016)]. Thus, there is the need to find treatments efficient in rescuing/preventing neurological manifestations, taking advantage of the window between the appearance of the first manifestations and neurological symptoms (Vroegindeweij et al., 2015; Pelucchi et al., 2018). The variable efficacy of ICT on neurological symptoms may depend on the ability of different chelators to cross the BBB, the timeliness of the treatment, the large heterogeneity of therapeutic protocols, and the unclear kinetics of iron chelators in the brain *in vivo* [reviewed in (Dusek et al., 2016)]. In the large majority, therapeutic efficacy was evaluated in a short time, and the few data available in long-term follow-up studies showed progression of neurological derangements (Pelucchi et al., 2018). ICT seems more promising if started before the onset of neurological symptoms, although the majority of the cases that remained asymptomatic have not yet reached the risk age [reviewed in (Dusek et al., 2016)]. Also, ICT must be often discontinued because of the aggravation of functional iron deficiency anemia, limiting the long-term therapy required to mobilize iron from the brain (Loreal et al., 2002; Mariani et al., 2004; Fasano et al., 2008; Finkenstedt et al., 2010). Due to the antioxidant properties of zinc sulfate and minocycline, the inhibitory effect on iron absorption of zinc sulfate, and chelating properties of minocycline iron, these have been proposed as alternatives to ICT when the latter must be discontinued, but the results, although promising, are limited to only two patients (Kuhn et al., 2007; Hayashida et al., 2016). To prevent tissue damage, antioxidants like vitamin E and C have been used along with ICT (Kuhn et al., 2007; Pelucchi et al., 2018).

**Table 1:** Therapeutic approaches for aceruloplasminemia.

**Iron chelation**		
*Deferoxamine*	*Deferasirox*	*Deferoxamine-Deferiprone*
Miyajima et al., 1997	Skidmore et al., 2008	Fasano et al., 2008
Yonekawa et al., 1999	Bethlehem et al., 2010	Mariani et al., 2004
Loreal et al., 2002	Roberti Mdo et al., 2011	Badat et al., 2015
Haemers et al., 2004	Suzuki et al., 2013	Bove and Fasano, 2015
Finkenstedt et al., 2010	Tai et al., 2014	Poli et al., 2017
Hida et al., 2010	Rusticeanu et al., 2014	Pelucchi et al., 2018
Pan et al., 2011	Lindner et al., 2015	
	Doyle et al., 2015	
*Deferiprone*	*Deferiprone-Deferasirox*	*Deferoxamine-Deferasirox*
Pelucchi et al., 2018	Pelucchi et al., 2016	Calder et al., 2017
	Pelucchi et al., 2018	
**Zinc administration**		
Kuhn et al., 2007		
**Minocycline administration**		
Hayashida et al., 2016		
**Iron chelation + Vitamin E and C**		
Kuhn et al., 2007		
Pelucchi et al., 2016		
Pelucchi et al., 2018		
**Iron chelation + Fresh Frozen Plasma**		
Logan et al., 1994		
Yonekawa et al., 1999		
Poli et al., 2017		
**Iron chelation + Fresh Frozen Plasma with high Cp level**		
*“under development”*		
**Enzyme Replacement Therapy**		
Harris et al., 1999*		
Zanardi et al., 2018*		
**Gene Therapy**		
*“desirable”*		

*^∗^Preclinical model.*

In some cases, ICT was combined with fresh-frozen plasma (FFP) administration. Due to the half-life of Cp (5.5 days) (Hellman and Gitlin, 2002), FFP administration could partially/temporarily restore circulating Cp. In two cases, the combined therapy improved neurological symptoms, visibly reducing brain iron deposition (Yonekawa et al., 1999; Poli et al., 2017). This implies that circulating Cp can enter the CNS and be functionally effective. Since physiological serum Cp concentration ranges between 21 and 54 mg/dL (Gibbs and Walshe, 1979; Gaasch et al., 2007), the selection of high-Cp-content FFP transfusions might be more effective, thereby advancing this therapeutic approach. A post-transfusion Cp level of 8–10 mg/dL, comparable to that of ACP heterozygotes, could be enough to rescue iron homeostasis as inferred by the absence of clinical symptoms in the vast majority of heterozygous subjects (Miyajima, 1993; Kono, 2013). Nevertheless, the risks associated with long-term repeated transfusion could limit this approach.

A neuroprotective effect of ceruloplasmin administration was reported in various pathological models (Ayton et al., 2013, 2014; Tuo et al., 2017; Zanardi et al., 2018). Indeed, it has been demonstrated that intraperitoneally administered Cp was able to enter the Cp-KO brain crossing the barrier systems (Ayton et al., 2013; Zanardi et al., 2018). The potential of the Cp-enzyme replacement therapy (ERT) in reducing neurological manifestation was recently demonstrated in the preclinical model of ACP (Zanardi et al., 2018). The ferroxidase activity was restored in Cp-KO brain, inducing reduction in iron deposition, rescue of neuronal loss, and amelioration of motor incoordination (Zanardi et al., 2018). The mechanism by which administered Cp crosses the brain barrier systems in Cp-KO, but not in wild-type mice, is not known. However, the iron accumulation found in the choroid plexus of Cp-KO mice suggested that Cp enters the brain likely through BCSFB impairment (Zanardi et al., 2018). A similar impairment would also explain the effectiveness of the FFP therapy in ACP patients. Cp administration also mobilizes iron and temporarily restores iron homeostasis in Cp-KO mice (Harris et al., 1999; Zanardi et al., 2018). Thus, ERT seems to be efficacious on both systemic and neurological symptoms, and may be a potential therapeutic opportunity in humans.

As for many monogenic rare diseases, the ideal ultimate cure consists in gene therapy approaches to correct the defect, but further studies and several years will be necessary before its clinical application.

## Conclusion

In conclusion, current therapies in ACP are effective in reducing systemic iron overload but have little or no effect on neurological symptoms. Their capability in improving pancreatic and retinal damage is unclear, and might have deleterious side effects that limit long-term therapy. FFP administration can be a suitable source of functional Cp, and although cost-benefit effects need to be further evaluated, can represent a step toward the development of more efficient therapies in ACP.

## Author Contributions

AP and MA contributed the same to conception and writing of the work.

## Conflict of Interest Statement

The authors declare that the research was conducted in the absence of any commercial or financial relationships that could be construed as a potential conflict of interest.
